# Hypoparathyroidism: what is the best calcium carbonate supplementation intake form?^☆^

**DOI:** 10.1016/j.bjorl.2017.10.010

**Published:** 2017-11-15

**Authors:** Loraine Gollino, Maria Fernanda Giovanetti Biagioni, Nathalia Regina Sabatini, José Vicente Tagliarini, José Eduardo Corrente, Sérgio Alberto Rupp de Paiva, Gláucia Maria Ferreira da Silva Mazeto

**Affiliations:** a Universidade Estadual Paulista “Júlio de Mesquita Filho” (Unesp), Faculdade de Medicina de Botucatu, Departamento de Medicina Interna, Botucatu, SP, Brazil; b Universidade Estadual Paulista “Júlio de Mesquita Filho” (Unesp), Faculdade de Medicina de Botucatu, Departamento de Oftalmologia, Otorrinolaringologia e Cirurgia de Cabeça e Pescoço, Botucatu, SP, Brazil; c Universidade Estadual Paulista “Júlio de Mesquita Filho” (Unesp), Instituto de Biociência, Departamento de Bioestatística, São Paulo, SP, Brazil

**Keywords:** Calcium, Calcium carbonate, Hypoparathyroidism, Phosphorus, Thyroidectomy, Cálcio, Carbonato de cálcio, Hipoparatireoidismo, Fósforo, Tireoidectomia

## Abstract

**Introduction:**

In hypoparathyroidism, calcium supplementation using calcium carbonate is necessary for the hypocalcemia control. The best calcium carbonate intake form is unknown, be it associated with feeding, juice or in fasting.

**Objective:**

The objective was to evaluate the calcium, phosphorus and calcium × phosphorus product serum levels of hypoparathyroidism women after total thyroidectomy, following calcium carbonate intake in three different forms.

**Methods:**

A crossover study was carried out with patients presenting definitive hypoparathyroidism, assessed in different situations (fasting, with water, orange juice, breakfast with a one-week washout). Through the review of clinical data records of tertiary hospital patients from 1994 to 2010, 12 adult women (18-50 years old) were identified and diagnosed with definitive post-thyroidectomy hypoparathyroidism. The laboratory results of calcium and phosphorus serum levels dosed before and every 30 min were assessed, for 5 h, after calcium carbonate intake (elementary calcium 500 mg).

**Results:**

The maximum peak average values for calcium, phosphorus and calcium × phosphorus product were 8.63 mg/dL (water), 8.77 mg/dL (orange juice) and 8.95 mg/dL (breakfast); 4.04 mg/dL (water), 4.03 mg/dL (orange juice) and 4.12 mg/dL (breakfast); 34.3 mg^2^/dL^2^ (water), 35.8 mg^2^/dL^2^ (orange juice) and 34.5 mg^2^/dL^2^ (breakfast), respectively, and the area under the curve 2433 mg/dL min (water), 2577 mg/dL min (orange juice) and 2506 mg/dL min (breakfast), 1203 mg/dL min (water), 1052 mg/dL min (orange juice) and 1128 mg/dL min (breakfast), respectively. There was no significant difference among the three different tests (*p* > 0.05).

**Conclusion:**

The calcium, phosphorus and calcium × phosphorus product serum levels evolved in a similar fashion in the three calcium carbonate intake forms.

## Resumo

**Introdução:**

No hipoparatireoidismo, a suplementação de cálcio com carbonato de cálcio é necessária para o controle da hipocalcemia. A melhor forma de ingestão de carbonato de cálcio ainda é desconhecida, seja concomitante com alimentação, no suco ou em jejum.

**Objetivo:**

Avaliar os níveis séricos de cálcio, fósforo e produto cálcio-fósforo em mulheres pós tireoidectomia por hipoparatireoidismo, após a ingestão de carbonato de cálcio em três formas diferentes.

**Método:**

Foi realizado um estudo cruzado em pacientes com hipoparatireoidismo definitivo, avaliados em diferentes situações (em jejum, com água, suco de laranja, café da manhã, após *washout* de uma semana). A revisão dos prontuários dos pacientes de um hospital terciário de 1994 a 2010 identificou 12 mulheres adultas (18-50 anos), diagnosticadas com hipoparatireoidismo definitivo pós-tireoidectomia. Os resultados laboratoriais dos níveis séricos de cálcio e fósforo foram mensurados antes e a cada 30 minutos durante 5 horas, após a ingestão de carbonato de cálcio (cálcio elementar 500 mg).

**Resultados:**

Os valores de pico máximo médio de cálcio, fósforo e produto cálcio-fósforo foram 8,63 mg/dL (água), 8,77 mg/dL (suco de laranja) e 8,95 mg/dL (café da manhã); 4,04 mg/dL (água), 4,03 mg/dL (suco de laranja) e 4,12 mg/dL (café da manhã); 34,3 mg^2^/dL^2^ (água), 35,8 mg^2^/dL^2^ (suco de laranja) e 34,5 mg^2^/dL^2^ (café da manhã), respectivamente, e a área sob a curva foi 2.433 mg/dL.min. (água), 2.577 mg/dL.min. (suco de laranja) e 2.506 mg/dL.min. (café da manhã), 1.203 mg/dL.min. (água), 1.052 mg/dL.min. (suco de laranja) e 1.128 mg/dL.min. (café da manhã), respectivamente. Não houve diferença significante entre os três diferentes testes (*p* > 0,05).

**Conclusão:**

Os níveis séricos de cálcio, fósforo e produto cálcio-fósforo evoluíram de forma semelhante nas três formas de ingestão de carbonato de cálcio.

## Introduction

Hypoparathyroidism (HypoPT) stems from dysfunctional production and/or secretion of active parathormone (PTH) by the parathyroid glands.^1^ HypoPT has many causes, and the most frequent are parathyroidectomy and thyroidectomy surgeries.^2^ After total thyroidectomy, the incidence of postoperative HypoPT ranges from 0.5% to 6.6%.^2^ However, incidences as high as 20% have been reported^3^, ^4^, ^5^, ^6^ depending on surgery extension and complexity, which are greater in malignant neoplasms, such as thyroid cancer, the main indication for total thyroidectomy.^7^, ^8^, ^9^ In these cases, central compartment neck dissection is a risk factor for permanent hypoparathyroidism.^10^

Permanent HypoPT after total thyroidectomy is characterized by persistent hypocalcemia and low or inappropriately normal levels of PTH for more than six months after surgery.^11^ Its treatment involves the supplement protocol of calcium and vitamin D^1^ to control the clinical manifestations of hypocalcemia, to maintain calcium (Ca) and phosphorus (P) levels, and adequate Ca × P product.^12^ For this treatment and in many other situations where Ca replacement is indicated, calcium carbonate (CaCO_3_) is the Ca salt most frequently prescribed because of its higher percentage of elemental Ca^13^ and better absorption in the normal or acidic pH of the stomach.^2^, ^14^, ^15^ Normal individuals should take CaCO_3_ with meals or a particular food to increase the bioavailability of the mineral.^16^ Yet, for HypoPT, where Ca supplement is associated with quality of life maintenance and patient survival, no studies have assessed the influence of CaCO_3_ intake protocol on certain parameters, such as calcemia and phosphatemia. Moreover, in normal individuals calcemia is rigorously controlled by a feedback system that involves many factors, especially PTH.^1^, ^17^, ^18^ This system maintains serum Ca levels constant, even when a Ca overload occurs, which impairs assessing the temporal effect of CaCO_3_ intake. In this sense, HypoPT patients could be a good model for assessing the real impact of CaCO_3_ intake protocols on calcemia and phosphatemia.

Thus, the present study investigated how the serum levels of Ca, P, and Ca × P vary over time after three different protocols of CaCO_3_ intake by women with permanent HypoPT secondary to total thyroidectomy.

## Methods

### Subject and methods

This is a crossover study that assessed three different intake protocols with a washout period of one week between assessments. All patients were submitted to the three intake protocols.

### Patients

The sample size was calculated to comply with the crossover design, considering a 10% difference between treatment means and a coefficient of variation of 10%.^19^ According to this analysis, the sample should have at least 12 individuals. Data were collected from patients submitted to total thyroidectomy secondary to differentiated thyroid carcinoma (DTC) between 1994 and 2010 at the Hospital das Clínicas, Faculdade de Medicina de Botucatu-UNESP. Twelve females aged 18–50 years were selected. These patients, who did not have other comorbidities, had been diagnosed with permanent HypoPT, defined as the presence of persistent hypocalcemia and low or inappropriately normal serum PTH levels for at least one year after total thyroidectomy.^4^ They were regularly followed at a outpatient clinic.

### Ethics, consent and permissions

This study was approved by the Research Ethics Committee of the institution, under protocol number 4332-2012, in accordance with the Helsinki Declaration of 1975, with approvement of Clinical Trial Registration (Number: 4332-2012) and all participants signed an informed consent form to participate in the study.

### Data collection

Tests were conducted to assess the serum levels of Ca, P and of the Ca × P product over time after three different CaCO_3_ supplementation protocols: after an overnight fast, taken with 200 mL of water; after an overnight fast, taken with 200 mL of orange juice SuFresh^®^ (Wow Indústria Comércio, Caçapava, Brazil); and taken with 200 mL of water right after breakfast (bread roll with margarine and sweetened coffee). The CaCO_3_ dose was 1282 mg (Oscal^®^, Sanofi Aventis, Suzano, Brazil), equivalent to 500 mg of elemental Ca, which is the dose habitually prescribed for HypoPT patients, who take 1–3 g of elemental Ca per day,^2^ averaging 1.5 g/day divided into two or three doses. The order of the protocols was varied by raffle to minimize the possibility of one influencing the other.

The baseline serum levels of Ca, P, magnesium (Mg), alkaline phosphatase (ALP), total proteins and fractions, PTH and 25-hydroxyvitamin D before CaCO_3_ administration were measured regardless before each protocol. After CaCO_3_ administration, the serum levels of Ca and P were measured every 30 min for 5 h (11 samples per participant).

### Statistical analyses

The serum Ca and P levels were expressed as mean and standard deviation. Independent samples were analyzed by analysis of variance (ANOVA), followed by the multiple comparison Tukey test for symmetric distribution, adjusting general linear models with Gamma distribution, followed by the multiple comparison Wald test for asymmetric data. Pearson's correlation was performed between the Ca area under the curve (AUC) and the serum levels of 25-hydroxyvitamin D, and between the Ca AUC and the age of the women. All analyses were performed by the statistical applications SAS for Windows^®^ version 9.3 and SigmaStat 3.5. The significance level was set at 5%.

## Results

### Cohort description

Females had a mean age of 43 years and most were white. The average time between thyroidectomy and the tests was 8.6 years. All patients were treated with CaCO_3_, with a mean elemental Ca intake of 856 mg, and most also took calcitriol (Table 1).

**Table 1:** General characteristics and effective treatment for chronic hypocalcemia of 12 patients with permanent hypoparathyroidism due to total thyroidectomy for differentiated thyroid carcinoma.

General characteristics	*n* = 12
Age (years)^a^	43.3 ± 7.3
Caucasion^b^	11 (91.7%)
Education attainment: secondary degree of secondary school^b^	4 (33.2%)
Various workers^b^	7 (58.3%)
Time after thyroidectomy (years)^a^	8.60 ± 5.4
CaCO_3_ supplement use^b^	12 (100%)
CaCO_3_ supplement dose (mg/day)^a^	2141 ± 1193
Elemental calcium intake (mg/day)^a^	856 ± 477
Calcitriol supplement use^b^	10 (83.3%)
Calcitriol supplement dose (μg/day)^a^	0.38 ± 0.18

a Values expressed as mean ± SD.

b *n* (%); *n*, number of patients; CaCO_3_, calcium carbonate.

### Temporal variation of serum calcium, phosphorus, and Ca × P

**Figure 1:**
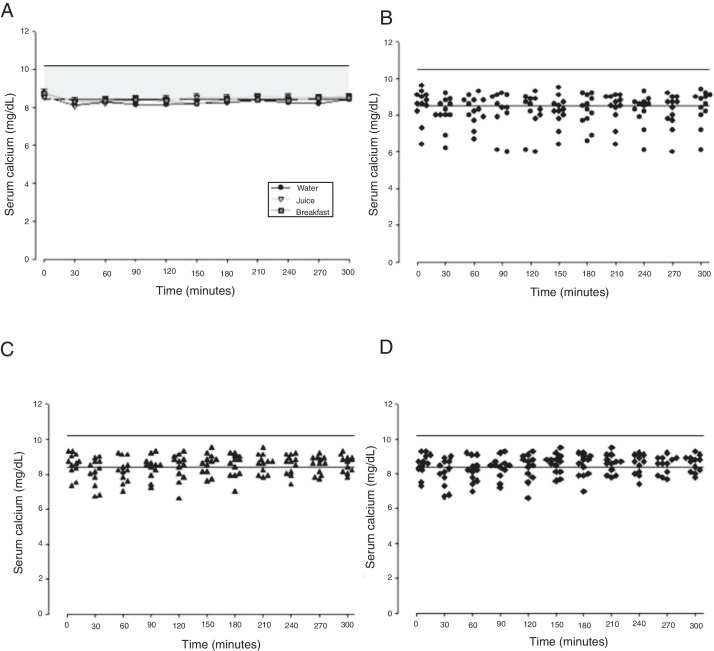
Evolution of the serum calcium after calcium carbonate supplementation according to the different intake forms. A, means and standard errors; B, C and D, serum calcium scatter plots in fasting with water, with orange juice and after breakfast, respectively.

**Figure 2:**
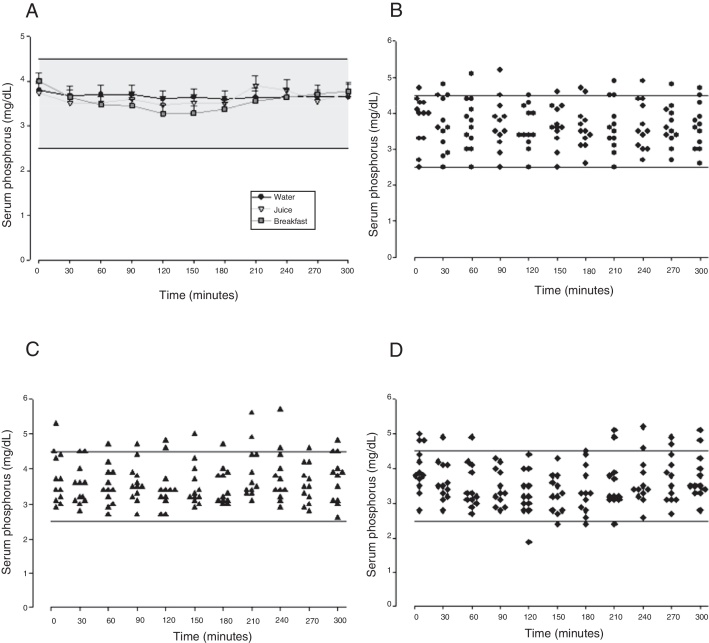
Evolution of serum phosphorus after calcium carbonate supplementation according to the different intake forms. A, means and standard errors; B, C and D, serum phosphorus scatter plots in fasting with water, with orange juice and after breakfast, respectively.

**Figure 3:**
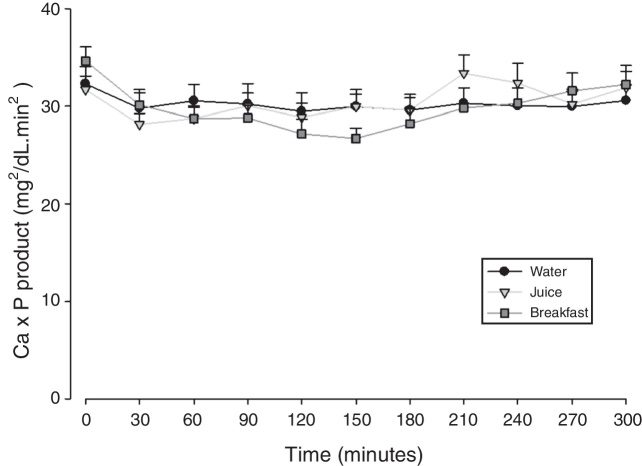
Evolution of the values of calcium × phosphorus product (Ca × P) expressed as mean standard error, after calcium carbonate supplementation according to the different intake forms.

The mean baseline hormone and biochemical levels did not differ significantly (*p* > 0.05) in the three CaCO_3_ intake protocols (Table 2). Calcemia and phosphatemia had similar curves regardless of CaCO_3_ intake protocol. The mean Ca levels were below the lower limit of normality, and the mean P levels were in the reference range (Figure 1, Figure 2). The Ca × P product remained below 55 mg^2^/dL^2^ at all times, and its temporal variation was similar in the three protocols (Fig. 3).

**Table 2:** Baseline biochemical and hormonal serum levels.

Serum	CaCO_3_ supplementation			*p*-value
	Water	Juice	Breakfast	
Calcium (mg/dL)	8.54 ± 3.32	8.54 ± 3.32	8.73 ± 3.32	0.809
Phosphorus (mg/dL)	3.8 ± 0.69	3.74 ± 0.72	4 ± 0.65	0.640
Magnesium (mg/dL)	1.93 ± 0.17	1.88 ± 0.13	1.92 ± 0.17	0.693
Total protein (g/dL)	6.84 ± 0.58	6.89 ± 0.55	7.05 ± 0.76	0.716
Albumin (g/dL)	3.93 ± 0.38	3.98 ± 0.24	3.98 ± 0.38	0.939
Globulin (g/dL)	2.91 ± 0.34	3.25 ± 1.00	3.08 ± 0.48	0.482
Alkaline phosphatase (U/L)	72.58 ± 12.2	74 ± 15.8	71.33 ± 12.7	0.840
25-hydroxyvitamin D (ng/mL)	32.19 ± 10.2	33.36 ± 11.6	32.87 ± 9.56	0.964
PTH (pg/mL)	12.36 ± 8.66	14.01 ± 9.63	12.46 ± 8.04	0.876
TSH (μLU/mL)^a^	1.21 ± 2.88	0.92 ± 2.01	0.9 ± 2.36	0.896
FT4 (ng/mL)	1.34 ± 0.28	1.35 ± 0.31	1.33 ± 0.38	0.990

Values expressed as mean ± SD. Statistical tests: ANOVA followed by Tukey.

a Adjustment in distribution range (asymmetric data); significance, *p* < 0.05. CaCO_3_, calcium carbonate; FT4, free thyroxine; TSH, thyrotropin; PTH, parathyroid hormone. Reference values: calcium 8.4–10.2 mg/dL; phosphorus 2.5–4.5 mg/dL; magnesium 1.6–2.3 mg/dL; total protein 6.3–8.2 g/dL; albumin 3.5–5 g/dL; globulin 1.4–3.2 g/dL; alkaline phosphatase 35–104 U/L; 25-hydroxyvitamin D 30–60 ng/mL; PTH 11–65 pg/mL; TSH 0.4–4 μLU/mL; FT4 0.8–1.8 ng/mL.

The means of the peak, time-to-peak, and AUC for serum Ca and P and Ca × P product did not differ by CaCO_3_ intake protocol (Table 3), even after adjusting the CaCO_3_, elemental Ca, and calcitriol doses (data not shown).

**Table 3:** Maximum peak, time to peak and area under the curve of serum calcium, phosphorus and area under the curve calcium × phosphorus product.

Serum		CaCO_3_ supplementation			*p*-value
		Water	Juice	Breakfast	
Calcium	Maximum peak (mg/dL)	8.63 ± 0.87	8.77 ± 0.55	8.95 ± 0.38	0.477
	Time to peak (min)	202.5 ± 85.9	182.5 ± 57.8	152.5 ± 94.5	0.326
	AUC (mg/dL min)	2433 ± 239	2577 ± 214	2506 ± 121	0.226
	Maximum peak (mg/dL)	4.04 ± 0.76	4.03 ± 0.69	4.12 ± 0.66	0.945
Phosphorus	Time to peak (min)	142 ± 92.1	135 ± 99.0	167 ± 107	0.706
	AUC (mg/dL min)	1203 ± 173	1052 ± 119	1128 ± 320	0.981
Ca × P product	Maximum peak (mg^2^/dL^2^)	34.3 ± 6.55	35.8 ± 7.34	34.5 ± 6.41	0.827
	Time to peak (min)	180 ± 85.9	187 ± 85.9	192 ± 100	0.944
	AUC (mg^2^/dL min^2^)	9038 ± 1645	8846 ± 1365	9094 ± 1095	0.900

Values expressed as mean ± SD.

Statistical tests: ANOVA followed by Tukey; significance: *p* < 0.05.

AUC, area under the curve; CaCO_3_, calcium carbonate; Ca × P product, product calcium × phosphorus.

The age [*r* = 0.063 (water), *r* = −0.14 (juice), *r* = 0.08 (breakfast)] and the serum concentrations of 25-hydroxyvitamin D [*r* = 0.18 (water), *r* = 0.28 (juice), *r* = 0.20 (breakfast)] did not correlate (*p* > 0.05) with the Ca AUC.

## Discussion

HypoPT may be a consequence of total thyroidectomy,^3^, ^20^ which is the most frequent treatment for DTC,^21^ a neoplasm whose incidence has increased considerably in the last years.^22^, ^23^ Patients with permanent HypoPT require lifelong treatment with Ca salts to control calcemia, phosphatemia, and the Ca × P product.^2^, ^12^, ^24^ CaCO_3_ is the most common salt used for this purpose because of its higher elemental Ca percentage^13^ with good absorption.^14^ Studies that assessed calcemia, phosphatemia, and Ca × P product in different CaCO_3_ intake protocols, in HypoPT patients after thyroidectomy, were not found.

This study compared three different protocols of CaCO_3_ supplementation, equivalent to 500 mg of elemental calcium. Serum Ca over time did not differ by protocol. The mean serum Ca levels remained in the lower limit of normality, as recommended for HypoPT patients.^12^ Calcemia of healthy women did not vary over time after CaCO_3_ supplementation.^25^ On the other hand, Ca serum of women with polycystic ovary syndrome increased significantly.^26^ However, comparison of the study results with individuals with normal PTH secretion is inappropriate. Additionally, although the therapeutic objectives seem to have been achieved, individual analysis of the three intake protocols showed that roughly 41% of serum Ca values were below the lower limit of normality. Hypocalcemia may have unknown health repercussions.

The calcemia peaks ranged from 8.6 to 8.9 mg/dL, and the times-to-peak ranged from 152 to 202 min, with an AUC of 2433–2577 mg/dL min regardless of CaCO_3_ intake protocol. The peaks remained in the lower half of normality, which is desirable in HypoPT patients because serum Ca in these patients should remain low, despite the adverse effects of hypocalcemia.^12^ These values differ from those reported by Tondapu and contributors^27^ who studied CaCO_3_ supplementation in patients submitted to the bariatric surgery Roux-en-Y and found a peak of 9.2 mg/dL, time-to-peak of 126 min, and AUC of 3240 mg/dL min. The different results are justified by the fact that both calcemia and AUC rely on PTH action, which was normal in the sample studied.^27^ PTH controls calcemia rigorously, as shown by a crossover study of healthy women that compared CaCO_3_ and placebo intakes and did not find differences in the Ca peak and AUC.^25^ Eventually, the age and vitamin D sufficiency of the patients could have influenced the results obtained. However, no significant associations were found between these parameters and Ca AUC.

The study time-to-peak means were higher than those reported elsewhere,^27^ which may also stem from low PTH. Still, Wang and contributors^28^ assessed healthy premenopausal women and found a Ca time-to-peak of 240 min, higher than the study time-to-peak. On the other hand, Heller and contributors^29^ assessed healthy postmenopausal women and found a time-to-peak of 174 min, similar to the study time-to-peak.

The phosphatemia of normal individuals has a circadian rhythm, with a nadir at around 10 in the morning and a peak at around 2 in the afternoon, generally ranging from 2.4 to 3.6 mg/dL.^30^ Phosphatemia is affected by food intake. Valderas and contributors^31^ assessed phosphatemia for 3 h after a standard meal and found mean phosphatemia values ranging from 3.1 to 3.5 mg/dL. At eight in the morning, time of the first blood collection, the participants’ phosphatemia ranged from 2.7 to 5.7 mg/dL. The last blood samples were collected at one o’clock in the afternoon, when normal individuals have a discrete elevation of serum phosphorus.^30^ Phosphatemia peak, time-to-peak, and AUC were similar in all three CaCO_3_ intake protocols, regardless of food intake. The mean peak values ranged from 4.03 to 4.12 mg/dL, time-to-peak ranged from 135 to 167 min, and AUC ranged from 1052 to 1203 mg/dL.min. Although mean phosphatemia was within the reference range, roughly 10% of the participants had P levels beyond the recommended limits, especially above, which may negatively impact their metabolic control. In HypoPT hyperphosphatemia is almost as harmful as hypocalcemia. Hyperphosphatemia is associated with lower bone resorption^32^ and calcification of the basal ganglia^33^ and coronary artery.^34^, ^35^ In normal individuals hyperphosphatemia decreases calcemia, which stimulates PTH secretion and consequently, increases calcemia. Thus, in HypoPT patients hyperphosphatemia worsens hypocalcemia even more because of PTH deficiency.^36^ Phosphatemia must be rigorously monitored to avoid significant fluctuations, since hypophosphatemia may also have negative effects, as it is associated, for example, with higher childhood mortality.^37^ Interestingly, because of hypocalcemia, CaCO_3_ may also be used for controlling hyperphosphatemia.^38^

In HypoPT the Ca × P product should stay below 55 mg^2^/dL^2^,^39^ to avoid precipitation of Ca–P complexes in soft tissues, such as basal ganglia, lens, and kidneys,^11^ and vascular calcification,^33^, ^40^, ^41^ especially in the coronary arteries.^35^ In addition to organic processes, neuropsychological disorders have been associated with changes in the Ca × P product.^42^ In untreated HypoPT calcemia decreases and phosphatemia increases, so the Ca × P product should not change. In fact, this product did not change in rats submitted to parathyroidectomy.^32^ Nonetheless, when these patients take Ca supplements to correct hypocalcemia, Ca × P product may increase. In the present study, all Ca × P product values were below the recommended upper limit, and the mean Ca × P product values were similar in the three CaCO_3_ intake protocols.

This study has some limitations, such as the small sample size. Nevertheless, the sample size was calculated statistically based on the study design. Another limitation is the relatively low Ca dose used (500 mg of elemental Ca), which may have contributed to the similar temporal variations of the study parameters in the three CaCO_3_ intake protocols. Still, other studies that used the same dose observed significant changes, with an increase in calcemia.^26^, ^29^, ^43^ Moreover, the study dose would be the recommended dose for HypoPT patients, given that the recommended 1–3 g dose of elemental Ca per day is divided into two or three doses.^2^ As a matter of fact, once the maximum intestinal solubility of CaCO_3_ is reached, higher doses would not be absorbed.^44^ Another limitation would be the evaluation of total serum Ca instead of ionized Ca, which is effectively active in blood. However, the measurement of total Ca is more available in clinical practice and, since there were no differences between serum albumin levels in the three situations evaluated, it was considered that this measure could be used.^2^ Despite the limitations, the present study is the first to assess the temporal variation of calcemia and phosphatemia after CaCO_3_ supplementation in HypoPT patients. Furthermore, this endocrine disorder may be a model for the assessment of CaCO_3_ per se, without the influence of PTH on the temporal variations of calcemia and phosphatemia. Since our findings suggest that better Ca absorption in HypoPT patients does not require taking the salt after meals, perhaps this conclusion could be extrapolated to other conditions that require Ca supplementation.

## Conclusion

The temporal variations of calcemia, phosphatemia, and the Ca × P product in women with permanent hypoparathyroidism secondary to total thyroidectomy are similar regardless of the CaCO_3_ supplementation protocol (water, juice or breakfast). Therefore, considering only calcemia and phosphatemia, these patients may take CaCO_3_ after an overnight fast with water or orange juice, or after breakfast.

## Funding

This work was supported by the 10.13039/501100003593National Council for Scientific and Technological Development (CNPq – Conselho Nacional de Desenvolvimento Científico e Tecnológico), no. 130424/2013-7, and had no influence on the design of the study, or on the collection, analysis, and interpretation of data, or on the writing the manuscript.

## Conflicts of interest

The authors declare no conflicts of interest.
